# Development of extracellular matrix supported 3D culture of renal cancer cells and renal cancer stem cells

**DOI:** 10.1007/s10616-018-0273-x

**Published:** 2018-12-31

**Authors:** Kamila Maliszewska-Olejniczak, Klaudia K. Brodaczewska, Zofia F. Bielecka, Wojciech Solarek, Anna Kornakiewicz, Cezary Szczylik, Camillo Porta, Anna M. Czarnecka

**Affiliations:** 1 Department of Oncology, Military Institute of Medicine, Szaserow 128, 04-141 Warsaw, Poland; 2 School of Molecular Medicine, Medical University of Warsaw, Zwirki i Wigury 61, 02-091 Warsaw, Poland; 3 Present Address: National Centre for Nuclear Research, Sołtana 7, 05-400 Otwock, Poland; 4 Department of Oncology, European Health Centre, Borowa 14/18, 05-400 Otwock, Poland; 5 Department of Internal Medicine and Therapeutics, University of Pavia, Pavia, Italy; 6 Division of Translational Oncology, IRCCS Istituti Clinici Scientifici Maugeri, Pavia, Italy

**Keywords:** Renal cancer, Cell culture, Stem cells, 3D culture, Xeno-free, Serum-free

## Abstract

Novel experimental conditions of cancer cell line culture have evolved throughout the recent years, with significantly growing interest in xeno-free, serum-free and three-dimensional culture variants. The choice of proper culture media may enable to mimic tumor microenvironment and promotion of cancer stem cells proliferation. To assess whether stem-like phenotype inducing media may be applied in renal cancer stem cell research, we performed a widespread screening of 13 cell culture media dedicated for mesenchymal cells, stem cells as well as mesenchymal stem cells. We have also screened extracellular matrix compounds and selected optimal RCC 3D—ECM supported culture model. Our results revealed that 786-O as well as HKCSCs cell line cultures in xeno-free media (NutriStem/StemXvivo) and laminin coated plates provide a useful tool in RCC cancer biology research and at the same time enable effective drug toxicity screening. We propose bio-mimic 3D RCC cell culture model with specific low-serum and xeno-free media that promote RCC cell viability and stem-like phenotype according to the tested genes encoding stemness factors including E-cadherin, N-cadherin, *HIF1*, *HIF2*, *VEGF*, *SOX2*, *PAX2* and *NESTIN*.

**Electronic supplementary material:**

The online version of this article (10.1007/s10616-018-0273-x) contains supplementary material, which is available to authorized users.

## Introduction

The vast majority of molecular cancer studies are conducted using immortalized cell lines cultured as two-dimensional (2D) monolayer on polystyrene surface, which does not reflect tumor in vivo structure and constrain stem-like phenotype (Bielecka et al. 2016; Cattin et al. 2018; Khawar et al. 2018; Boghaert et al. 2017). On the contrary, it was shown that cell physiology, metabolome, gene expression or morphology resembling that in solid tumor tissue may be maintained, if cells are cultured in specific culture conditions, including appropriate growth factor supplementation, extracellular matrix (ECM) support, oxygen tension and three-dimensional (3D) structures development (Bielecka et al. 2016). In the light of these data 3D in vitro cell cultures have been widely utilized in cancer research as culture promoting propagation of cancer stem cells subpopulation (Maliszewska-Olejniczak et al. 1817; Bussolati et al. 2008) and a model more closely mimicking in vivo tumor characteristics. In particular it was shown that cancer stem cells that are found in 3D cultures express not only stemness genes encoding SRY-Box 2: SOX2, Octamer-binding transcription factor 4: OCT-4, or Homeobox transcription factor Nanog, but also pro-angiogenic growth factors like vascular endothelial growth factor: VEGF (Bielecka et al. 2016).

**Fig. 1:**
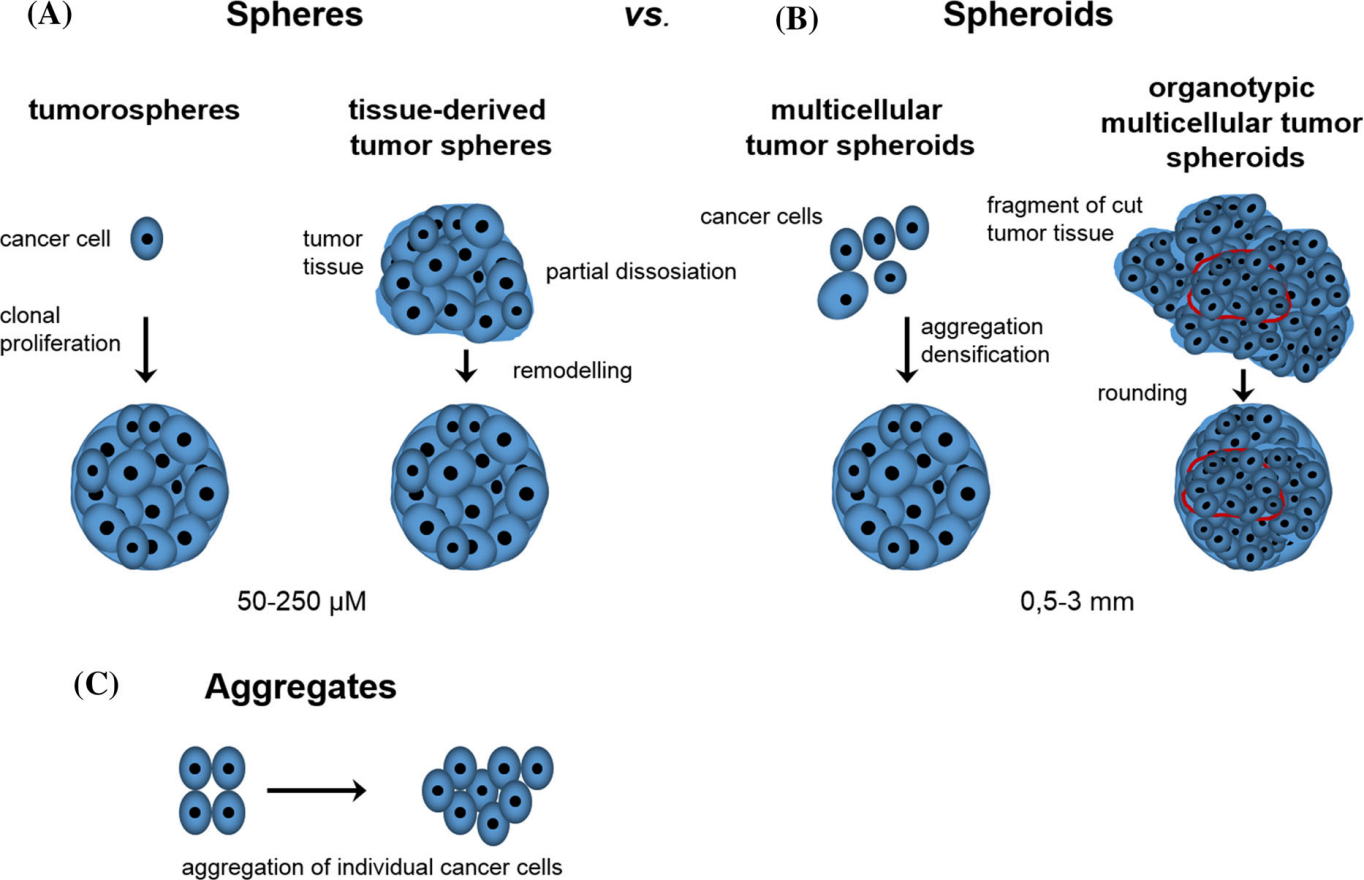
Schematic representation of possible 3D cell morphology. **a** Tumorospheres: are developed after proliferation of cancer cells in low-adherent conditions in stem cell medium, cancer cells are fused together in spherical structure and individual cells are difficult to distinguish. Tissue-derived tumor spheres: are established by mechanical separation and partial dissociation of tumor tissue, enabling maintaining cell–cell, cell–matrix interactions in non-adherent conditions. **b** Multicellular tumor spheroids: clusters of cancer cells starting from single cell suspensions generated in non-adherent conditions, aggregating and compacting to form spherical structure. Organotypic multicellular tumor spheroid: tumor tissue is cut and grow in non-adherent conditions, rounding and forming spherical structure. **c** Aggregates: individual cancer cells aggregate, attached to another and form clusters (not spherical) composed of cells easily distinguishable

In the present study 3D cultures were classified according to its spatial structure. In particular 3D sphere-like structures fulfilled the following criteria in accordance with the applicable classification: had a smooth, curved shape, with the presence of cancer cells and their capability to be maintained as free-floating cultures, not attached to anything and able to move freely (Weiswald et al. 2015). Among sphere-like structures most common are (1) spheres (Fig. 1a: tumorospheres and tissue-derived tumor spheres) and (2) spheroids (Fig. 1b: multicellular tumor spheroids and organotypic multicellular spheroids).

*Spheres* are determined as cancer cells growing in nonadherent conditions, forming 3D clusters (Cao et al. 2011). Tumorospheres represent free-floating spheres of cancer stem cell culture in a serum-free medium supplemented with growth factors and were firstly described in brain tumors by Singh et al. (2003) and Weiswald et al. (2015). Only cancer stem cells (or stem-like cells) with tumor initiation, self-renewing and propagation potential as well as lineage tracing capacity can form 3D spheres in culture. Since sphere-forming cells are stem-like cells, they also have the ability to differentiate into all of the non-stem-like cell subpopulations found in the initial cell culture and as a result tumorosphere is a mixture of CSCs and differentiated cells. At the same time tissue-derived tumor spheres are established by mechanical separation and incision from tumor tissue, enabling maintaining cell–cell contact of cancer cells.

The term *spheroid* is used to describe clusters of cancer cells starting from single cell suspensions generated in nonadherent conditions (Yamada and Cukierman 2007). Research on multicellular tumor spheroids (MCTS) where cells are more differentiated than in flat monolayer cultures, was initiated in the early 70s by Sutherland’s group (Sutherland et al. 1971). In comparison to MCTS, organotypic multicellular spheroids (OMS) are obtained by the cutting of cancer tissues in nonadherent environment and are resembling the tumor microenvironment, thus preserving the integrity of the tumor-stroma interplay (Bjerkvig et al. 1990; Vaira et al. 2010).

It is worth mentioning that except spherical cancer models, other 3D structures of cultured cells like *aggregates, colonies* and *organoids* can be formed. Interestingly, compact spherical cultures can form free bundles of cancer cells and then they are termed as aggregates (Fig. 1c) (Ivascu and Kubbies 2006). Moreover, single cancer cells are able to proliferate and then form colonies in soft agar which enables to discriminate transformed from non-transformed cells (Macpherson and Montagnier 1964). Finally, organoid form (meaning mini-organ-like) should be referred to normal cells and tissue cultured in 3D systems (Weiswald et al. 2015; Clevers 2016).

In order to maintain in culture tumor-derived cancer cells including cancer stem-like cells and propagate cancer spheroids or spheres, it is important to select specific growth media with serum (referred as to serum-containing) or without (serum-free) and with or without animal-derived products (xenogeneic or xeno-free) (Usta et al. 2014). Serum-free media contain minimal amount of essential components and xeno-free (XF) medium should not contain animal-derived additives, however may contain human-derived components. Xeno-free and serum-free media can preserve in vivo-like phenotype of numerous cell lines including neurons, fibroblast and cancer cells with special emphasis on primary cancer stem cells derived from glioblastoma (Usta et al. 2014). Interestingly, it has been demonstrated that the *xeno-free medium* system preserves morphology of human embryonic stem cells (hESCs) in an undifferentiated condition for a long time (Zhang et al. 2016). Moreover MSCs expanded in XF/SF conditions showed significantly higher yield in comparison with serum-containing medium (Weiswald et al. 2015; Swamynathan et al. 2014). In the light of this trend towards elimination of media containing serum and animal-derived components (xenogeneic) is currently observed in the in vitro studies. It is widely recognized and many projects have adopted these approaches to study cancers, including kidney cancers (Schmeichel and Bissell 2003). Specific conditions were recently characterized promoting RCC cell viability using specific serum-free and xeno-free medium (Cattin et al. 2018). The authors have developed more controlled and defined biomimic cell culture system, useful in down-stream applications.

Among available 3D in vitro assays, spherical cancer models have recently been described in cancer stem cell research with special emphasis on different variants (Khawar et al. 2018). However, appearing nomenclature in the literature is not consistent and confusing when distinguishing different models of cancer spheres which seems to be critical in usage of spheroid monocultures in anti-cancer drug testing therapies. Therefore our study is focused on standardization of the concepts of 3D structures in serum and xeno-free cultures.

Based on our recent analyses (Balachander et al. 2015) we selected both clear cell and papillary RCC cell lines including: 769-P (primary tumor origin, clear-cell renal cell carcinoma—ccRCC), 786-O (primary tumor origin, ccRCC), Caki-2 (primary tumor origin, papillary RCC—pRCC), ACHN (metastasis, pleural effusion, pRCC) and HKCSCs (human kidney cancer stem-like cells of papillary RCC origin and applied xeno-free and serum-free culture models (Khan et al. 2015). We believe that screening is a powerful and useful tool for researchers aiming at obtaining fast-growing, reliable cell culture which would clearly reconstitute in vivo tumor features. Until now, no clear guidelines on how cancer stem-like cell colonies morphologically differ and which structure more adequately represents the stem-like cell rich structures in RCC culture model has been published.

The main goal of the study was to establish the best RCC cell culture system for small-molecule activity exploration. We describe the process of screening for optimal 3D culture conditions for selected RCC cell lines along with verification of the stem-like phenotype preservation in these 3D culture systems. Tested media (Table S3) were dedicated for cancer stem cell, mesenchymal stem cell (MSCs), as well as iPS/ES, since these media are expected to promote and maintain 3D cell culture growth. Cell lines tested covered both primary tumor derived and metastatic tumor derived cell lines. By selecting media that promote 3D structure formation and extracellular matrix that further stabilize 3D structures we aim to propose optimal culture system to grow RCC stable cancer cell lines including its stem-like cell subpopulation (HKCSCs) for in vitro testing.

## Materials and methods

### Cell lines

All cells used in the research were frozen after 1st passage and kept in liquid nitrogen. The stocks used in this research were independently defrozen in the years 2014–2016 and used for no more than 6 months. In total, 769-P, 786-O, Caki-2 (all from ATCC) and HKCSCs (Celprogen, Inc., Cat. No. 36117-44, Bielecka et al. 2017) cell lines were used. During subsequent stages of the research, chosen cell lines were analyzed. Characterization of cell lines used in the study as well as their origin and reason of their analysis is shown in Table S4. ATCC cell lines were tested and authenticated, assuring of their identity on the basis of morphology tests, karyotyping and PCR, free from intra- and interspecies contamination. All cell lines cultured in the laboratory are screened for *Mycoplasma* contamination on regular basis with Mycoplasma Detection Kit (Jena Bioscience, PP-401L) and only negative passages were used for presented experiments.

### Culture media

Cells were seeded on 24-well plates (standard tissue-culture treated, Sigma Aldrich, Cat. No. CLS3738-100EA, Laminin-coated, Corning, 354412 or Poly-d-Lysine-coated, Corning, 354619) in density of 10,000 cells/well and cultured in media 1–14 for 6–14 days (depending on confluence reach). All media initially used in the research are enumerated and described in Table 2 and S3. Specific number is dedicated to specific cell medium throughout the whole study. The cells were cultured in media accordingly with manufacturers’ instructions. Selected cell line-media sets were chosen as best for 3D culture based on confluence reach velocity and 3D structures morphology.

### Cells isolation

Cells were cultured as described above and aggregates were collected from wells, washed with PBS (pH = 7.2) and incubated for 10 min with Accutase solution (Biowest) to obtain single cell suspensions. Then, cells were washed and re-suspended in different buffers (depending on assay) and used in further analyses.

### Cells visualization

Cells were visualized using inverted microscope with Olympus camera UC30 (serial no. 14310982) and Olympus Entry Cell Sense 1.8.1. software (serial no. PY8HDQECP6Q, core version XV 3.8.). The size of three-dimensional structures was measured with CellSens software (Olympus); area of representative aggregates/colonies/spheres was marked and calculated accordingly to magnification of the microscope.

### Drug treatment

After formation of 3D structures, in vitro drug treatment was applied; Temsirolimus (TEM, Pfizer) or Epirubicin (EPI, Accord) were added in different concentrations. Cells were cultured for additional 3 days to monitor the changes in sphere morphology. Standard 2D culture was prepared in parallel.

### Cells viability analysis

Cells were suspended in 0.2 ml of serum-free DMEM and viability was measured with Muse Count &Viability Assay Kit (Merck Millipore, MCH100102) according to manufacturer’s instructions. Briefly, 50 μl of cell suspension was mixed with 450 μl of viability stain and incubated for 5 min. Then, 2000 cells were acquired on Muse Cell Analyzer (Merck Millipore, 0500-3115) and percentage of live and dead cells was measured.

### Cell cycle analysis

Cells were pelleted to remove PBS, suspended by vortexing and pipetting and then 1 ml of 70% ice-cold ethanol (Chempur, 113964200) was slowly added. Cells were incubated at − 20 °C for at least 3 h. Before analysis, alcohol was removed completely by two PBS washes and then 100 μl of Muse Cell Cycle reagent (Merck Millipore, MCH100106) was added for 30 min. For acquisition, additionally 150 μl of PBS was added. At least 2500 cells were acquired and analysed for percentage of cells in G0/G1, S and G2 phases, basing of DNA content.

### RNA isolation

Total RNA was isolated with Cells-to-Ct kit (Ambion, AM1728); 5000 cells were suspended to remove excess PBS and 50 μl of DNase containing Lysis buffer was pipetted to the sample. After 5 min incubation, reaction was stopped by gently in-mixing 5 μl of Stop Solution. Resulting RNA isolates were stored in − 80 °C and then used for reverse transcription using above mentioned kit. cDNA was obtained by adding 2X RT Buffer and 20X RT Enzyme mix to 10 μl of RNA isolates; reaction was adjusted to 25 μl with water and run for 60 min in 37 °C, followed by 5 min inactivation in 95 °C in a LifeECO thermalcycler (Bioer, BYQ6078). Resulting cDNA was stored in − 20 °C and used in gene expression analysis.

### Gene expression analysis

Real-time PCR was performed using TaqMan Gene Expression Master Mix (Applied Biosystems, 4369010) with multiplex TaqMan primer/probe sets (Applied Biosystems; listed in Table 3) in 20 μl reactions in triplicates with 2 μl of 2X diluted cDNA reaction as a template. Two gene TaqMan sets were used in each reaction; compatibility of the kits was confirmed before experiments and only kits that gave same signal when run in single or double reactions were used in the study. Also, -RT (minus reverse transcription) negative controls without reverse transcriptase were prepared. Reactions were run in 8-well strips on LightCycler96 machine (Roche, 05815916001). Data were calculated with 2^(−Delta delta C(T))^ method, with normalisation to geometrical mean expression of *PPIA* and *GUSB* as house-keeping gene controls (Khawar et al. 2018) and represented as fold change in comparison to standard monolayer culture (RPMI with FBS on tissue culture treated plates).

### Statistical analysis

All experiments were performed in at least three repetitions, one representative study is shown. Results are presented as mean ± SD from triplicates (3 wells). Differences between groups were determined using Student T test and considered significant if *p* < 0.05 and marked as * on graphs.

## Results

### Choice of optimal 3D-promoting culture conditions

We screened 13 culture media among those available on the market in order to establish an optimal cell culture conditions promoting 3D growth of RCC cells in a reproducible manner (Table 1). Tested media (listed and characterized in Table S3, Figure S1) consisted of cancer stem cell dedicated formulations but also mesenchymal cell and iPS/ES since these media are expected to promote and maintain 3D cell culture growth.

**Table 1:** Workflow of performed experiments throughout the study, accordingly with the subsequent numbers

Experiment performed	Materials used	Purpose	Main results
1. Media testing	769-P, 786-O, Caki-2, ACHN, HKCSCs Media 1–14 accordingly with Table 2	Establish best culture media for each cell line for 3D structures formation	Presented in: Table 1, Figs. 1, 2, Supplementary Figure 1a Best sets cell line-medium for 3D structures: HKCSCs-14, 769-P-4, 786-O-14, Caki-2-4, ACHN-4,11
2. Viability and cell cycle analysis	786-O and media chosen in step 1	Confirm the microscope observations and the effect of 3D culture on cell growth	Presented in Fig. 3a, b
3. Gene expression analysis	786-O and media chosen in step 1	Verify the effect of 3D structure formation on stem-like phenotype	Presented in Fig. 4 and Table 3
4. Drug treatment	786-O and media chosen in step 1	Validate the model	Presented in Fig. 5

**Table 2:** Summary of results of cell cultures in different media

	1	2	3	4	5	6	7	8	9	10	11	12	13	14
	MSCs	MSCs	SCs	MSCs	SCs	SCs	MSCs	HEK	HEK	MSCs	SCs	MSCs	N/A	SCs
	SF	RS	SF	SF	S	SF	S	S	S	RS	SF	S	S	S
	X	X	X	XF	X	X	X	X	X	X	X	X	X	XF
	+/−	–	+	+	–	?	–	?	?	–	+	–	–	+
HKCSCs	+	–	+/−	x	–	–	–	x	x	–	++	–	–	+++
786-O vhl mut	–	–	–	–	x	–	x	x	x	x	+	x	–	++
769-P vhl mut	x	–	x	+	–	x	–	x	x	–	x	–	–	+/−
ACHN vhl wt	–	–	x	+/−	–	–	–	x	x	–	++	–	–	x
Caki-2 vhl wt	x	–	x	+/−	–	–	–	x	x	–	–	–	–	x

*HEK* medium for HEK cells, *S* medium contains serum, *SF* serum free medium, *RS* reduced serum medium, *X* xenogeneic medium, *XF* xeno-free medium

+ Visible 3D structures of diameter > 300 μm, suggesting tumor-like hypoxia inside the structure

+/− Visible small 3D structures or both 2D and 3D cell culture

– No visible 3D structures visible, but cells growing (i.e. in 2D or in small microaggregates)

X lack of cell growth in general

**Table 3:** List of paired TaqMan assays

Gene (FAM stain)	Assay ID	Gene (VIC stain)	Assay ID
*HIF1*	Hs00153153_m1	*HIF2*	Hs01026149_m1
*VEGF*	Hs00900055_m1	*PAX2*	Hs01565576_m1
*VHL*	Hs00184451_m1	*CDH2*	Hs00983056_m1
*CDH1*	Hs01023894_m1	*C*-*MET*	Hs01565576_m1
*CD133*	Hs01009257_m1	*NANOG*	Hs04399610_g1
*NESTIN*	Hs04187831_g1	*CD105*	Hs00923996_m1
*SOX2*	Hs01053049_s1	*OCT4*	Hs04260367_gH
*PPIA*	Hs01565699_g1	*GUSB*	Hs00939627_m1

**Fig. 2:**
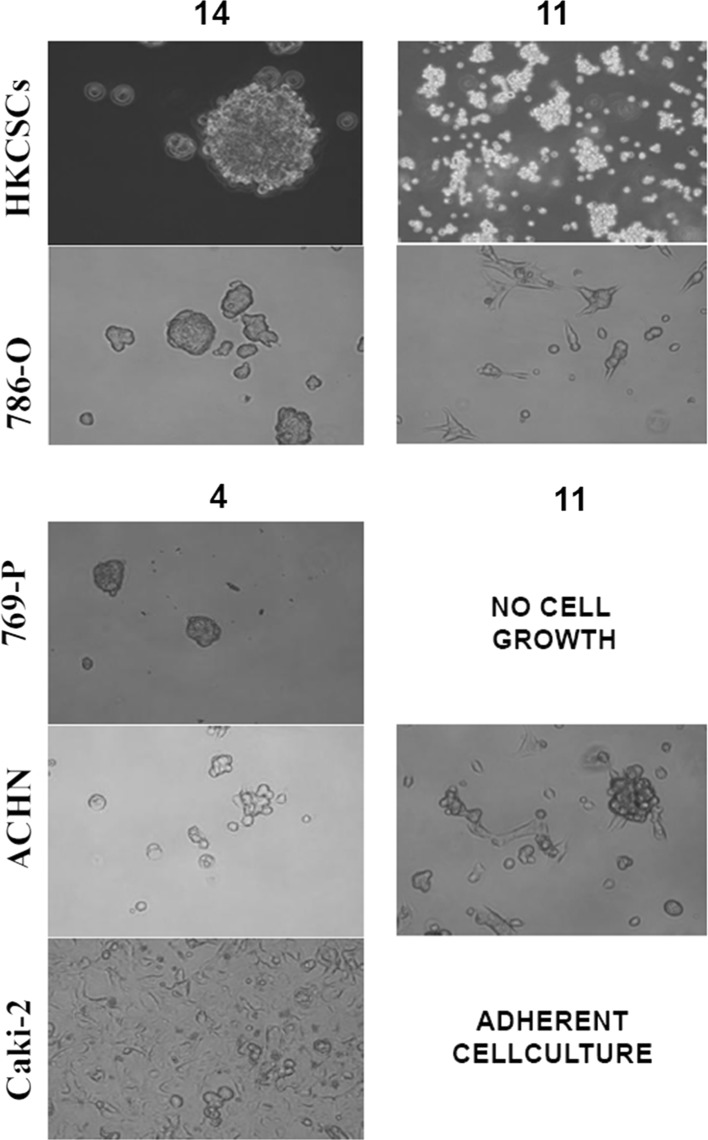
Representative photos of cell morphology observed in culture during media screening

Screened RCC cell lines, both VHLwt and mutated (Tables S1, S2), proliferated rapidly in multiple media dedicated to stem cells and mesenchymal stem cells (MSCs), also in serum reduced conditions, however not always the predicted culture morphology (2D or 3D) was obtained (Figure S2, S3, Table S4). In general serum concentration reduction did not significantly reduce RCC cell proliferation while serum deprivation did. The outcome of culture was strongly cell line dependent, however it was not correlated with the VHL mutation status as we used both VHL mutated and wild type cells (Table S2). The results of media screening are summarized in Table 2 with representative photos of morphology of obtained structures on Fig. 2. None of the tested cell lines survived in serum-free and remained mostly adherent in MSCs dedicated media (Table 2 and Fig S1), therefore these media were excluded from further analysis.

**Fig. 3:**
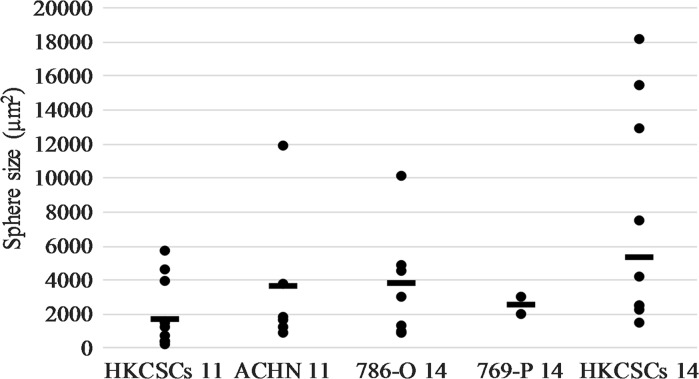
The sizes of three-dimensional structures measured with CellSens software (Olympus); area of representative aggregates/colonies/spheres in specific cell line/media was marked and calculated accordingly to magnification of the microscope

HKCSCs, primary cells enriched in stem-like cells by culture selection, were more prone to grow in non-adherent morphology, also in MSCs formulations as they formed 3D structures in 4 out of 13 tested media (Figure S2). With established cell lines, the success rate of three-dimensional growth was lower; usually only 1 or 2 medium formulation was capable to sustain 3D growth of cells. The most effective media in promoting sphere-like structures were medium 11 (StemXVivo; Semi-solid medium formulated and optimized for tumorsphere formation, supplemented with epithelial to mesenchymal transition (EMT) inducing supplement) and medium 14 (NutriStem; Xeno-free, feeder-free medium with reduced growth factor concentration) (Figure S3). Medium 11 promoted formation of large 3D structures of ACHN cells, and smaller 3D structures of 786-O. In medium 11 HKCSCs developed aggregates rather than colonies. On the other hand, HKCSCs growth rate was faster than of other 3D RCC cultures in medium 11. The largest 3D structures of stem cells—HKCSCs—were observed in medium 14 and this were spheroids and colony-like structures (Fig. 2). 3D structures of HKCSCs were not uniform and were rather similar to cell aggregates, which however may be useful i.e. in drug testing since the proliferation of these cells were of very high rates. The size of all obtained structures was at least 2000 μm^2^ (Fig. 3, Figure S4) and largest for HKCSCs. For further testing we chose 786-O cells cultured in media 11-StemXVivo and 14-Nutristem, to verify that 3D growth correlates with stem-like properties.

### Effect of 3D growth on cell survival and cycling

**Fig. 4:**
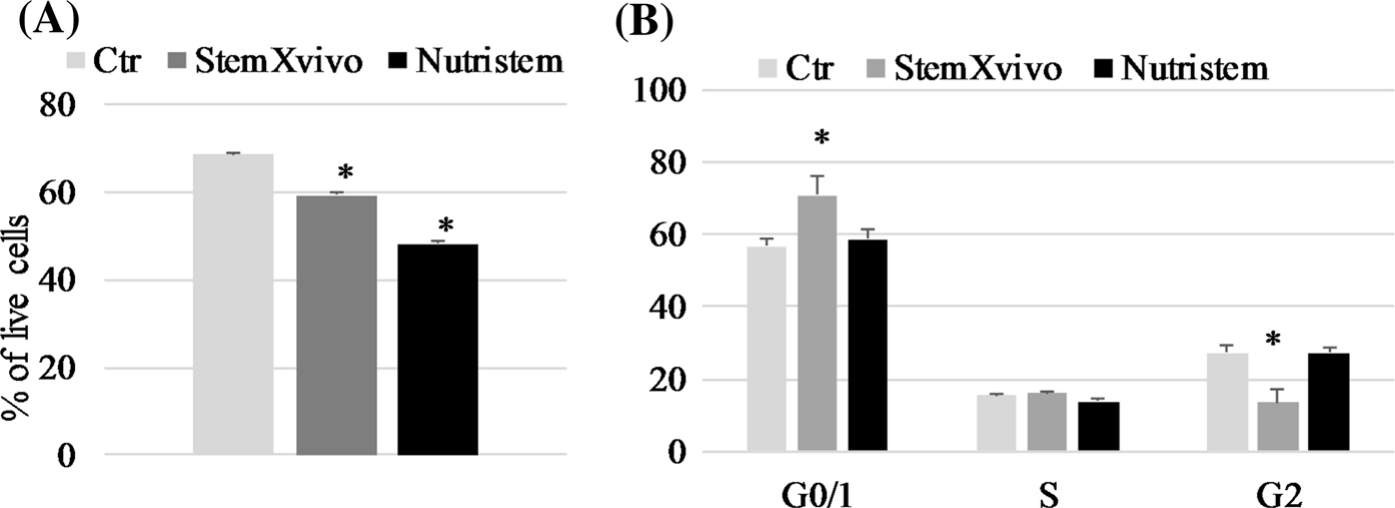
Changes of **a** viability and **b** cell cycle distribution of 786-O cells cultured in RPMI, StemXvivo or NutriStem media. *p < 0.05 in T student test versus Ctr variant

Both 3D-promoting media significantly reduce cell viability (Fig. 4a), probably due to selective conditions; cells that are not able to form spheres undergo cell death. Additionally to decreased cell viability, 786-O cells cultured in StemXvivo medium presented altered cell cycle distribution; increased percentage of cells in G0/1 phase was observed which coincided with reduced amount of G2 cells (Fig. 4b).

### 3D culture significantly modulate RCC cell stem-related gene expression

**Fig. 5:**
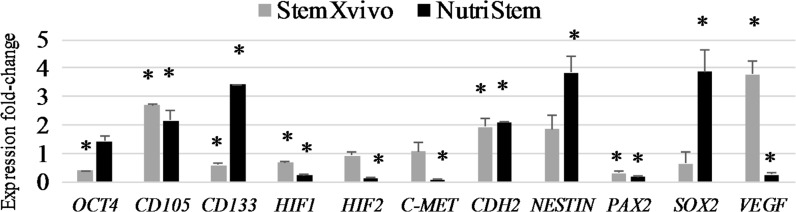
Changes in gene expression of 786-O cells cultured in StemXvivo or NutriStem media. Data expressed as fold change in comparison to standard monolayer culture. * *p* < 0.05 in T student test for the fold-change, ud-under detection

Selected media differentially altered gene expression in RCC 786-O cells (Fig. 5). In comparison to standard monolayer culture, Nutristem medium upregulated expression of stem-related genes encoding factors *SOX2* and *NESTIN*. *NANOG* and *C*-*KIT* stem markers were not detected in this cell line (data not shown). Also signs of epithelial–mesenchymal transition (EMT) were observed as *CDH2* (gene encoding N-cadherin) expression was enhanced by these culture conditions while *CDH1* (gene encoding E-cadherin) dropped to undetectable levels (but it was detected in monolayer culture). Also the level of putative RCC stem cell markers was enhanced, namely *CD105* and *CD133*. At the same time, *PAX2* kidney development regulator dropped in Nutristem 3D structures, suggesting cell differentiation. Also, *c*-*Met* oncogene expression was reduced, although it is suggested as a CSCs marker in other cancers. Despite creation of dense spheres in Nutristem, expression of both *HIF1* (Hypoxia-inducible factor 1) and *HIF2* was reduced when compared to epithelial growth. However, 786-O is a *vhl* mutant therefore HIF signaling might be deregulated in these cells. At the same time gene encoding pro-angiogenic factor *VEGF* was also down-regulated in response to formation of 3D structures. Culturing 786-O cells in StemXvivo medium effected in different gene expression pattern, probably due to big contribution of epithelial-like growth. Most CSCs related factors, apart from *CD105* and *Nestin*, were down-regulated as in comparison to control medium (RPMI with 10% FBS). *HIF1* level also dropped, with no effect in *HIF2* but *VEGF* production was up-regulated (Fig. 5). The effect of 3D growth in selected media on gene expression is summarized in Table 4.

**Table 4:** Summary of the effect of chosen culture conditions on characteristic gene expression

786-O	CSCs factors	CD105	CD133	EMT	HIF	VEGF
StemXvivo	↓	↑	↓	+	=	↑
NutriStem	↑	↑	↓	+	↓	↓

### 3D growth modifies drug response

**Fig. 6:**
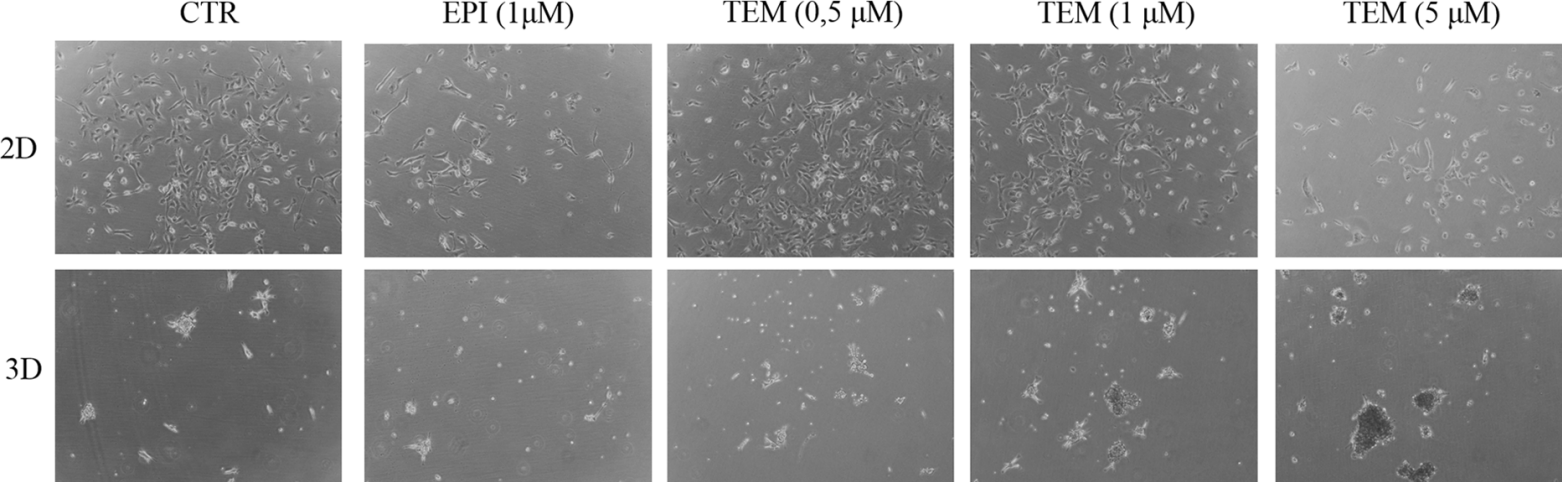
Effect of 3D growth of 786-O reaction to in vitro drug treatment with TAM and Epi. 3D cells was obtained in StemXvivo medium

Regarding 2D conditions, we have confirmed in stem cell promoting conditions, what has been shown in previous regular cell culture studies (Bielecka et al. 2017; Gotink et al. 2011), that kinase inhibitors effectively inhibit RCC cancer cell proliferation in higher concentrations (Fig. 6, top; Figure S5), including both typical anti-RCC compounds sunitinib, and sorafenib (Figure S5, S6) and in typical normoxic (21% O2, as well as intra-tumoral like hypoxic conditions (Figure S7). Temsirolimus (TEM) is currently used as 1st line treatment for ccRCC patients with poor predicted outcome. Treating 786-O cells with temsirolimus and epirubicin (typical cytostatic drug) was aimed to test whether our established 3D culture model would better mimic in vivo conditions to study ‘molecular resistance’ to therapies in vitro.

Temsirolimus in concentrations up to 1 µM in 2D culture was not effective in 786-O cells growth and proliferation limitation, but its cytostatic effect on monolayer cell culture was visible from the concentration of TEM from 5 µM (Fig. 6). Epirubicin, a typical cytostatic drug, visibly lowered 786-O viability in a lower dose (1 µM). The same effect was observed for EPI in 3D cell culture. The opposite effect was noticed when activity of TEM was taken into account in 3D cell culture. 3D structures of 786-O were visibly of bigger size and diameter when TEM was added. It suggests an opposite effect than observed in 2D culture and presumably it better reflects in vivo conditions, making 3D model more suitable for testing antiangiogenic agents than standard 2D in vitro cultures.

### Addition of surface modification

**Fig. 7:**
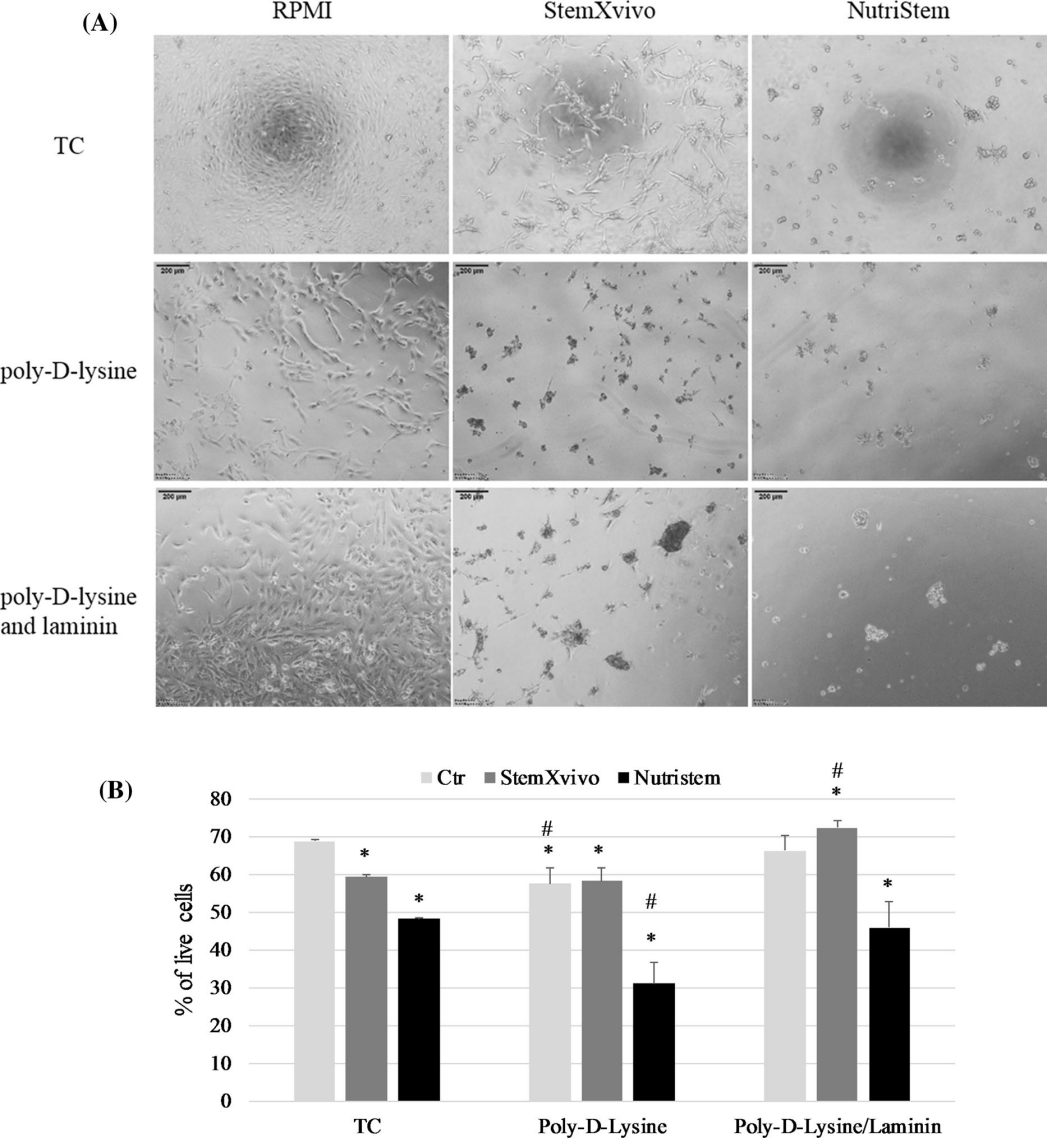
Effect of surface coating on 786-O growth; **a** morphology of cells, **b** changes of viability of 786-O cells cultured in RPMI, StemXvivo or NutriStem media and coated plates. * *p* < 0.05 in T student test versus TC Ctr variant, #*p < 0.05 in T student test versus TC variant of medium

Additional approach to promote formation of 3D structures in vitro may was culture on ECM coated plates. As shown on Fig. 7 A both poly-d-lysine and poly-d-lysine/laminin covered culture plates reduced adherence and epithelial morphology of 786-O cells in xeno-free media. Although spheres grew larger in 3D media, monolayer growth rate in standard FBS-containing medium was slower on coated surfaces in comparison to standard tissue-culture plates. However, poly-d-lysine/laminin coating increased cell viability in StemXvivo medium in comparison to standard tissue-culture treated plates in this medium (Fig. 7b). Both poly-d-lysine and poly-d-lysine/Laminin coated surface reduced the cell adherence in StemXvivo and NutriStem media with no visible differences in morphology of cells cultured in FBS-supplemented RPMI. However, the growth rate of 786-O cells cultured in modified surface was reduced in all used media.

**Fig. 8:**
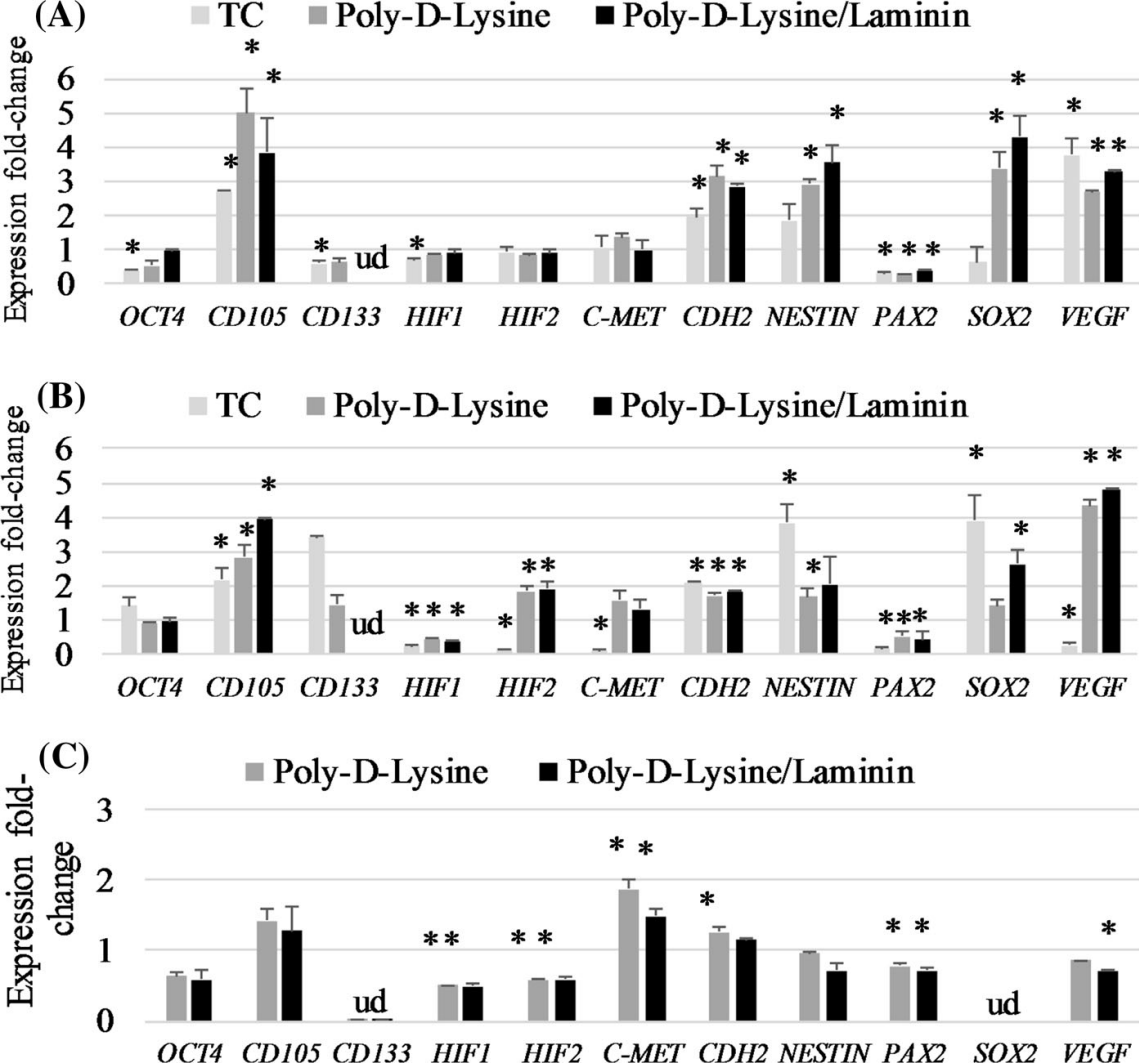
Changes in gene expression of 786-O cells cultured in different conditions: **a** in StemXvivo medium and coated plates, **b** in NutriStem medium and coated plates or **c** in RPMI/FBS medium in NutriStem medium and coated plates. Data expressed as fold change in comparison to standard monalayer culture. *p < 0.05 in T student test for the fold-change, ud-under detection

Culturing cells in matrix-coated plates in 2D medium only weakly changed expression of tested genes (Fig. 8). Both applied types of coatings remarkably reduced level of *CD133, CDH1* (E-cadherin) (to undetectable levels) and *SOX2* (to undetectable levels). However, simultaneous *CDH2* (N-cadherin) up-regulation was observed only in cells cultured on poly-d-lysine. Interestingly, 786-O cells cultured on both types of coatings were characterized by reduced expression of *HIF1* and *HIF2* (Fig. 8c). Use of combination of sphere-medium and coated plate induced the gene expression profile similar to the conventional TC plates in a given medium. StemXvivo medium on the plates covered with poly-d-Lysine and poly-d-Lysine with Laminin reduced the expression of *OCT*-*4* and *HIF1* (Fig. 8a). However, this effect was slightly weaker than the standard TC plates. The use of coated plates further strengthened the stimulant impact of StemXvivo on the expression of *CD105, NESTIN* and *CDH2*, but weakened the effect on *OCT*-*4, HIF1, PAX2* and *VEGF*. However, the use of both types of surfaces with StemXvivo medium, unlike the polystyrene plates, increased expression of *SOX2* (Fig. 8a). In general, standard tissue culture treated surface used with 3D promoting media seem to be sufficient to maintain three-dimensional growth of tested RCC cells and induce features of stem-like phenotype.

## Discussion

The growth of renal cancer and renal cancer stem cells in in vitro conditions requires optimal stability between signals mediating proliferation, cell survival and self-renewal. For in vitro application of RCC, the use of completely defined conditions and elimination of animal-derived materials from the culture is recommended (Krawczyk et al. 2018). Therefore, we aimed to develop the most efficient animal serum free culture system for RCC experiments through comparing available stable RCC cell lines and verifying different culture media. Subsequently, we have tested the ability to produce spheres and 3D spheroids in RCC culture—the evidence of stem cell phenotype through regulation of stemness genes and drug resistance capability. We aim to develop cell culture model with more in vivo like conditions in terms of growth factors derived stimulation.

It has previously been demonstrated that cancer stem cells can be found in renal cell carcinoma by culturing RCC cell line derived 3D structures defined as spheres (Zhong et al. 2010). Sphere culture model most often represents spontaneous in vivo aggregation of cancer cells (Clevers 2016) with most commonly used multicellular tumor spheroid culture obtained by culture of cancer cell lines under nonadherent conditions. Tumorospheres represent culture of cancer stem cell established in a serum-free ‘stem cell’ medium supplemented with growth factors (Usta et al. 2014). Tumorospheres are formed due to cancer stem cells properties and are ideally created by clonal CSCs proliferation. Structures derived from aggregation of multiple clonal cells are not spheres which originate from one singe cancer stem cell and should be avoided. Firstly, single cell suspension should be cultured at low densities in specific serum-free medium in low attachment polystyrene. This enables proliferation of clonal non-adherent spherical clusters (Schmeichel and Bissell 2003). It is important that medium should not be supplemented with FBS or any other serum but rather with factors enhancing ‘stemness’ features like basic fibroblast factor (bFGF) or epidermal growth factor (EGF) with hormone additives like insulin, hydrocortisone and progesterone depending on the cancer type as described before (Bielecka et al. 2016; Weiswald et al. 2015). These compact structures can be maintained in culture from 5 days until 2 months. Low adherent cancer cell subpopulations—cells that detach after short trypsinization—are enriched in tumorigenic cancer stem-like cells. These cells express metastatic and epithelial-to-mesenchymal transition genes (Khan et al. 2015). This trypsin-sensitive subpopulation displays CSC-like phenotype—high ALDH activity, expression of stem cell surface markers and transcription factors, pluripotency and EMT associated genes including *SNAIL*, *SLUG*, *VIMENTIN*, *N*-*CADHERIN (CDH2)*, *CXCL10*, *OCT*-*4* and *BMI1*. Moreover trypsin-sensitive subpopulation possesses clonogenicity and high self-renewal potential (Khan et al. 2015). To culture single-cell derived spheres (tumorospheres), cell aggregation must be prevented, and cell-seeding density must be carefully controlled (Zhang et al. 2016) as it is cell line-specific, as we have previously shown for Caki-2, ACHN and HKCSC cell lines (Vaira et al. 2010; Swamynathan et al. 2014). HKCSCs were prone to form 3D structures in media 1,9,11,14—both serum-free and with serum—spheres and colonies. At the same time (tumor)spheres and spheroids were created by all tested RCC cell lines: 769-P in media 1 and 4; 786-O and ACHN—in media 11 and 14; Caki-2—in media 3 and 4. All spheroids and tumorospheres developed in stem-cell dedicated media, both in xenogenic and xeno-free conditions. This data confirms that for culture of 3D cancer structures, media of various components, including with/without serum and xenogeneic components may be recommended.

In the current study, we have established and characterized 3D in vitro models of renal cancer cells and renal cancer stem-like cells in serum- and xeno-free cultures on the basis of the phenotypic and molecular features (gene expression profiles) of obtained cultures. In the study presented by Lee et al. (2013) the expression of several cell adhesion markers (E-cadherin—CDH1, and N-cadherin—CDH2), intermediate filament proteins (pan-cytokeratin and vimentin), and β-catenin, a protein involved in Wnt signaling was tested under 3D conditions in epithelial ovarian cancer (EOC). Our studies have shown that the gene expression profiles changes in 3D models in comparison with monolayer cultures thus resembling the in vivo situation. In fact, we have shown that usage of Nutristem medium upregulated expression of stem-related genes encoding factors like *SOX2* and *NESTIN* in 786-O cells. Similar effects were observed in 3D cultures of adenocarcinomic human alveolar basal epithelial cells tested by Xue et al. (2015) and in adenoid cystic carcinoma shown by Liu et al. (2014).

Hypoxia-inducible factors (HIFs) signaling pathways have been studied recently as extremely attractive targets for therapeutic treatments (Myszczyszyn et al. 2015). Reduction of HIF activity in CSCs may promote differentiation, but at the same time disruption of the CSC niche which may lead to loss of stemness characteristics thus enabling response to standard chemo- and radiotherapy, leading to lower relapse rates (Myszczyszyn et al. 2015). We have observed reduced levels of *HIF1* and *HIF2* factors when spheres were formed in Nutristem medium. In another medium, StemXvivo, we have observed the opposite effect—downregulation of the same factors, with no effect on *HIF2*. In 786-O cells cultured in matrix coated plates, we have obtained reduced levels of *HIF1*and *HIF2* factors. Our findings have shown, that the use of sphere forming medium (StemXvivo) and poly-d-lysine coated plates with Laminin combination reduces the expression profile of genes encoding *OCT*-*4, HIF1* and *PAX2* and at the same time increases the expression profile of SOX2. The opposite effects have been observed for HIF1 signaling factor in breast cancer cells in 3D scaffolds (Balachander et al. 2015).

Promotion of stem cell proliferation is a derivative of their ability to survive in anchorage-independent conditions. If cancer (also RCC) cells are cultured in suspension, bulk non-stem cells are more likely to undergo anoikis, while stem-like cells survive and proliferate thus forming spheres. As such, the formation of spheres from RCC cancer cells (cell lines and primary cultures) can be used to identify cells with these stem-like characteristics (Zhang et al. 2016; Swamynathan et al. 2014; Zhong et al. 2010). Finally multicellular RCC spheroids may be important as 3D cell culture models for drug screening in the context of 3D cell–cell and cell–matrix interactions as well as in radio- and chemoresistance. Spheroids may also be used in studies of tumor growth and proliferation, immune system interactions, extracellular matrix remodeling and angiogenesis. Laminins have previously been shown important in kidney development and RCC progression, which is in consistence with our results or RCC cells growth promotion (Hirschhaeuser et al. 2010). Especially larger spheroids resemble heterogeneous tumors in vivo through limited supply of oxygen, nutrients and metabolites, and forming a necrotic core (Vinci et al. 2012). Moreover, spheroids represent expression profile of cancer cells which is closely similar to in vivo conditions in comparison to 2D cultures (Friedrich et al. 2009). Such culture models offer beneficial insights that help to bridge the gap between in vitro and in vivo models. ECM in cell culture may in fact function as ECM proteins in RCC tumors, where it impact progression by binding and regulating the activity of growth factors, including angiogenic VEGF. ECM proteins, including tested by us laminin, have been previously shown to induce proliferative signaling in cancer cells. This further confirm the need of multi-factorial cell culture model including growth factor and extracellular interactions regulation.

Spheroids also harbor potential to contribute to either eliminate poor drug candidates at the pre-animal and pre-clinical testing and to identify promising drugs targets as well as those which would fail in classical 2D cell assays (Gassenmaier et al. 2013). The good example of this phenomenon was presented in current study, where 3D model enabled to show opposite effects of drug treatment to that observed in 2D cultures. In 2D cultures, which do not closely mimic tumor microenvironment and cell–cell interactions typical for spheroidal in vivo tumors are not found, tested drugs inhibited flat, adherent cell culture growth in low concentrations (up to 5 µM TEM). However in 3D cell culture the opposite effect has been observed: low TEM concentrations made RCC cells even more aggressive than prior to treatment. Such phenomenon has been already described in the literature (Paez-Ribes et al. 2009). Although Temsirolimus used in this study for RCC cells treatment assessment is actually an antiangiogenic agent—an mTOR kinase inhibitor targeting endothelial cells, it should be noted that targeted therapy was already reported to also have direct cytostatic effect on RCC cells (Bielecka et al. 2017; Gotink et al. 2011). What is more, in the 3D model we can possibly observe other than cytostatic effect of kinase inhibitors on RCC cells. Thus 3D culture may enable to qualify the potential drug for futher animal studies as it better mimics tumor microenvironmental reality and may serve as better in vitro model. With more biomimic cell culture system unnecessary animal experiments could be obeyed.

## Conclusions

1. MesenCult SF a xeno-free and serum-free medium dedicated to 3D culture of mesenchymal stem cells, induces rapid proliferation of RCC cells.
2. Stemness inducing media: MesenCult SF, Stem Pro MSC, Cancer Stem Premium Medium and Nutristem XF/F promote RCC cells to grow both in 2D and 3D.
3. Xeno-free and serum-free media: Nutristem XF/F, MesenCult SF and StemXvivo allowed RCC 3D structures formation.
4. Most effective RCC 3D structures formation is obtained using xeno-free and xenogeneic media on laminin coated plates.
5. 3D structures formation influence RCC cells and RCC-CSCs cycling.
6. 3D culture significantly modulated RCC cell expression of ‘stemness’ genes encoding: *E*-*cadherin, N*-*cadherin, HIF1, HIF2, VEGF, SOX2, PAX2* and *NESTIN*.
7. ECM attachment only slightly optimize the 3D model; it influenced RCC cell hypoxia response gene expression and stem-like phenotype.
8. 3D growth modified kinase inhibitor but not cytostatic drugs response.

Summarizing, we have shown how different types of RCC cells grown in 3D conditions may represent more physiological interactions with extracellular matrix and neighbouring cells, we have tested different conditions, how each cell line can adapt to different medium and how it changes expression profile of specific markers. We recommend 786-O cell line as well as HKCSC culture in xeno-free media (NutriStem/StemXvivo) and laminin coated plates which provide a useful tool in RCC cancer biology research and at the same time enable effective drug toxicity screening. Proposed model better mimics in vivo tumor microenvironment and thus may become a tool for future clinical studies.

## Electronic supplementary material

Below is the link to the electronic supplementary material.

Supplementary material 1 (DOCX 4854 kb)

## References

[CR1] Balachander GM, Balaji SA, Rangarajan A, Chatterjee K (2015). Enhanced metastatic potential in a 3D tissue scaffold toward a comprehensive in vitro model for breast cancer metastasis. ACS Appl Mater Interfaces.

[CR2] Bielecka ZF, Maliszewska-Olejniczak K, Safir IJ, Szczylik C, Czarnecka AM (2016). Three-dimensional cell culture model utilization in cancer stem cell research. Biol Rev Camb Philos Soc.

[CR3] Bielecka ZF, Malinowska A, Brodaczewska KK, Klemba A, Kieda C, Krasowski P, Grzesiuk E, Piwowarski J, Czarnecka AM, Szczylik C (2017). Hypoxic 3D in vitro culture models reveal distinct resistance processes to TKIs in renal cancer cells. Cell Biosci.

[CR4] Bjerkvig R, Tønnesen A, Laerum OD, Backlund EO (1990). Multicellular tumor spheroids from human gliomas maintained in organ culture. J Neurosurg.

[CR5] Boghaert ER, Lu X, Hessler PE, McGonigal TP, Oleksijew A, Mitten MJ, Foster-Duke K, Hickson JA, Santo VE, Brito C, Uziel T, Vaidya KS (2017). The volume of three-dimensional cultures of cancer cells invitro influences transcriptional profile differences and similarities with monolayer cultures and xenografted tumors. Neoplasia.

[CR6] Bussolati B, Bruno S, Grange C, Ferrando U, Camussi G (2008). Identification of a tumor-initiating stem cell population in human renal carcinomas. FASEB J.

[CR7] Cao L, Zhou Y, Zhai B, Liao J, Xu W, Zhang R, Li J, Zhang Y, Chen L, Qian H, Wu M, Yin Z (2011). Sphere-forming cell subpopulations with cancer stem cell properties in human hepatoma cell lines. BMC Gastroenterol.

[CR8] Cattin S, Ramont L, Ruegg C (2018). Characterization and in vivo validation of a three-dimensional multi-cellular culture model to study heterotypic interactions in colorectal cancer cell growth, invasion and metastasis. Front Bioeng Biotechnol.

[CR9] Clevers H (2016). Modeling development and disease with organoids. Cell.

[CR10] Friedrich J, Seidel C, Ebner R, Kunz-Schughart LA (2009). Spheroid-based drug screen: considerations and practical approach. Nat Protoc.

[CR11] Gassenmaier M, Chen D, Buchner A, Henkel L, Schiemann M, Mack B, Schendel DJ, Zimmermann W, Pohla H (2013). CXC chemokine receptor 4 is essential for maintenance of renal cell carcinoma-initiating cells and predicts metastasis. Stem Cells.

[CR12] Gotink KJ, Broxterman HJ, Labots M, de Haas RR, Dekker H, Honeywell RJ, Rudek MA, Beerepoot LV, Musters RJ, Jansen G, Griffioen AW, Assaraf YG, Pili R, Peters GJ, Verheul HM (2011). Lysosomal sequestration of sunitinib: a novel mechanism of drug resistance. Clin Cancer Res.

[CR13] Hirschhaeuser F, Menne H, Dittfeld C, West J, Mueller-Klieser W, Kunz-Schughart LA (2010). Multicellular tumor spheroids: an underestimated tool is catching up again. J Biotechnol.

[CR14] Ivascu A, Kubbies M (2006). Rapid generation of single-tumor spheroids for high-throughput cell function and toxicity analysis. J Biomol Screen.

[CR15] Khan MI, Czarnecka AM, Helbrecht I, Bartnik E, Lian F, Szczylik C (2015). Current approaches in identification and isolation of human renal cell carcinoma cancer stem cells. Stem Cell Res Therapy.

[CR16] Khawar IA, Park JK, Jung ES, Lee MA, Chang S, Kuh HJ (2018). Three dimensional mixed-cell spheroids mimic stroma-mediated chemoresistance and invasive migration in hepatocellular carcinoma. Neoplasia.

[CR17] Krawczyk KM, Matak D, Szymanski L, Szczylik C, Porta C, Czarnecka AM (2018). Culture in embryonic kidney serum and xeno-free media as renal cell carcinoma and renal cell carcinoma cancer stem cells research model. Cytotechnology.

[CR18] Lee JM, Mhawech-Fauceglia P, Lee N, Parsanian LC, Lin YG, Gayther SA, Lawrenson K (2013). A three-dimensional microenvironment alters protein expression and chemosensitivity of epithelial ovarian cancer cells in vitro. Lab Invest.

[CR19] Liu LJ, Zhang J, Xiao ZF, Dai B, Sun MY, Chen L, Chen B (2014). Three-dimensional collagen scaffold enhances the human adenoid cystic carcinoma cancer stem cell and epithelial-mesenchymal transition properties. J Biomed Mater Res Part B Appl Biomater.

[CR20] Macpherson I, Montagnier L (1964). Agar suspension culture for the selective assay of cells transformed by polyoma virus. Virology.

[CR21] Maliszewska-Olejniczak K, Brodaczewska KK, Bielecka ZF, Czarnecka AM (1817). Three-dimensional cell culture model utilization in renal carcinoma cancer stem cell research. Methods Mol Biol.

[CR22] Myszczyszyn A, Czarnecka AM, Matak D, Szymanski L, Lian F, Kornakiewicz A, Bartnik E, Kukwa W, Kieda C, Szczylik C (2015). The role of hypoxia and cancer stem cells in renal cell carcinoma pathogenesis. Stem Cell Rev.

[CR23] Paez-Ribes M, Allen E, Hudock J, Takeda T, Okuyama H, Vinals F, Inoue M, Bergers G, Hanahan D, Casanovas O (2009). Antiangiogenic therapy elicits malignant progression of tumors to increased local invasion and distant metastasis. Cancer Cell.

[CR24] Schmeichel KL, Bissell MJ (2003). Modeling tissue-specific signaling and organ function in three dimensions. J Cell Sci.

[CR25] Singh SK, Clarke ID, Terasaki M, Bonn VE, Hawkins C, Squire J, Dirks PB (2003). Identification of a cancer stem cell in human brain tumors. Cancer Res.

[CR26] Sutherland RM, McCredie JA, Inch WR (1971). Growth of multicell spheroids in tissue culture as a model of nodular carcinomas. J Natl Cancer Inst.

[CR27] Swamynathan P, Venugopal P, Kannan S, Thej C, Kolkundar U, Bhagwat S, Ta M, Majumdar AS, Balasubramanian S (2014). Are serum-free and xeno-free culture conditions ideal for large scale clinical grade expansion of Wharton’s jelly derived mesenchymal stem cells? A comparative study. Stem Cell Res Therapy.

[CR28] Usta SN, Scharer CD, Xu J, Frey TK, Nash RJ (2014). Chemically defined serum-free and xeno-free media for multiple cell lineages. Ann Transl Med.

[CR29] Vaira V, Fedele G, Pyne S, Fasoli E, Zadra G, Bailey D, Snyder E, Faversani A, Coggi G, Flavin R, Bosari S, Loda M (2010). Preclinical model of organotypic culture for pharmacodynamic profiling of human tumors. Proc Natl Acad Sci USA.

[CR30] Vinci M, Gowan S, Boxall F, Patterson L, Zimmermann M, Court W, Lomas C, Mendiola M, Hardisson D, Eccles SA (2012). Advances in establishment and analysis of three-dimensional tumor spheroid-based functional assays for target validation and drug evaluation. BMC Biol.

[CR31] Weiswald LB, Bellet D, Dangles-Marie V (2015). Spherical cancer models in tumor biology. Neoplasia.

[CR32] Xue G, Ren Z, Grabham PW, Chen Y, Zhu J, Du Y, Pan D, Li X, Hu B (2015). Reprogramming mediated radio-resistance of 3D-grown cancer cells. J Radiat Res.

[CR33] Yamada KM, Cukierman E (2007). Modeling tissue morphogenesis and cancer in 3D. Cell.

[CR34] Zhang Y, Sun B, Zhao X, Sun H, Cui W, Liu Z, Yao X, Dong X (2016). Spheres derived from the human SN12C renal cell carcinoma cell line are enriched in tumor initiating cells. J Exp Clin Cancer Res.

[CR35] Zhong Y, Guan K, Guo S, Zhou C, Wang D, Ma W, Zhang Y, Li C, Zhang S (2010). Spheres derived from the human SK-RC-42 renal cell carcinoma cell line are enriched in cancer stem cells. Cancer Lett.

