# Supplementary material for: Development of extracellular matrix supported 3D culture of renal cancer cells and renal cancer stem cells
Source: Cytotechnology. 2018 Dec 31;71(1):149–63. doi: 10.1007/s10616-018-0273-x (PMC6368519; doi:10.1007/s10616-018-0273-x)

Supplementary materials

# RCC cell line mutation analysis

- 1. **DNA isolation method**

Total genomic DNA was isolated from cells cultured in T75 flasks in standard RPMI medium (no 13) with Genomic Mini kit (A&A Biotechnology). Upon 80% confluence cells were detached with Accutase (Biowest), washed twice in PBS, suspended in 100 μl of Tris buffer and lysed with 200 μl Lysis Buffer in the presence of Proteinase K for 20 min in 37°C. After Proteinase K inactivation in 85°C for 5 min, samples were centrifuged and DNA-containing supernatant was bound on a column. After washing, DNA was eluted with warm Tris buffer. The concentration and purity of DNA were determined by measuring the absorption at 260 nm and 280 nm in a Multiskan™ GO Microplate Spectrophotometer (Thermo Fisher Scientific). DNA was stored in 4°C until further analyses.

- 1. ***vhl* sequencing method**

Extracted DNA was subjected to PCR reactions with specific primers listed in Table S1 with TaqNova polymerase kit (DNA Gdansk) according to manufacturer’s instructions in 25 μl with cycling conditions: initial denaturation for 5 min in 94°C and 35 cycles of 94°C for 30 s, 58°C for 30 s, 72°C for 30 s, followed by 72°C elongation for 5 min in Life Eco thermalcycler (BioEr). Obtained products were sequenced by an outsource company (Genomed) in both directions with the use of previously mentioned PCR primers (with the exception of vhl2-forward; see Table S1). Obtained sequences were aligned with reference sequence of human *vhl* gene with GeneDoc software to identify point mutations. If mutations were present, cell line DNA was additionally analyzed by an outsource company (Genomed) with diagnostic genetic test for von Hippel Lindau syndrome, to confirm the mutation.

**Table S1**. Sequences of primers used in PCR and sequencing (*- primer used in sequencing only).

| Exon number | Forward primer | Reverse primer |
| --- | --- | --- |
| 1 | CGCGTTCCATCCTCTAC | ATGTGTCCTGCCTCAAG |
| 2 | GGGGAAATGGAGAAAATAGG *AAATAGGTGCCCTGACTC | GCTTTTGAGACACCATAACA |
| 3 | TAGTACAGGTAGTTGTTGGC | ACACATTTTAAGGTCCCCTC |

DNA sequencing confirmed *vhl* mutations in 786-O and 769-P cells and other cell lines showed no mutations in tested genes as shown in Table S2.

**Table S2.** Mutations in *vhl* and *c-met* genes detected in studied cell lines.

| Cell line | Detected mutations |
| --- | --- |
| 786-O | *vhl* exon 1: NM_000551.3 c311 (delG)  vhl exon 2: SNP rs1678607 (T>G), NM_000551.3 c341 (delC) |
| 769-P | *vhl* exon 2: SNP rs1678607 (T>G), rs115744107 (G>A), rs9819196 (G>A)  *vhl* exon 3: NM_000551.3 c539 (T>A), no rs |

# Media used for RCC cell growth analysis

**Table S3.** Media used for optimal RCC culture development characterization.

MSCs (mesenchymal stem cells), SCs (medium for stem cells other than mesenchymal stem cells), S (medium with serum), OCs (medium for cells other than stem), SF (serum-free medium), RS (reduced serum medium), 3D (medium for three-dimensional culture, enables formation of tumorspheres/spheres/ aggregates/colonies etc.), 2D (adherent cell culture), 2D+3D (adherent cell culture with colony forming units), XF (xeno-free), X (including xeno-derived components). Regarding xeno-derived components: if no information is included by the manufacturer, and at the same time manufacturer provides information on other xeno-free media by indicating this fact, the media without description on xeno-derivatives is judged as one with xeno-derived components and marked as X*. XF* describes medium 8 as animal-protein free, but no proof that medium is xeno-free in total is available.

| *No.* | *Name* | *Company* | *Specification per manufacturer* | *Category* |
| --- | --- | --- | --- | --- |
| 1 | STEM PRO MSC SFM™ Serum-Free Human Mesenchymal Stem Cell Culture Medium | Invitrogen (cat. no. A1033201) | Medium formulated for the growth and expansion of human mesenchymal stem cells (MSCs); enables growth and increased consistency of MSCs compared to classical serum-supplemented medium (DMEM + 10% FBS). Adherent cell culture, but colony forming units (CFU) are visible. | MSCs, SF, 2D+3D, X* |
| 2 | MesenPro RS™ Medium | Thermo Fisher Scientific (cat. no. 12746012) | Reduced serum (2%) medium specifically formulated to support the growth of MSCs. No gene expression changes. Adherent cell culture. Possible 3D structures when combined with scaffold. | MSCs, RS, 2D, X* |
| 3 | Cancer Stem Premium™ Medium | ProMab (cat. no. 20141-500) | Serum-free medium optimized for the growth and selection of spheroid-forming cancer stem cells (CSCs). Tumorspheres may form. As a result of our research mostly cell aggregates were visible (Caki-1). | SCs, SF, 3D, X* |
| 4 | MesenCult™-SF Culture Kit | STEMCELL Technologies  (cat. no. #05429) | Standardized, serum-free system for the culture of human MSCs. Clusters or aggregates should form; it has been achieved during 2^nd^ day of cell culture (Caki-1, 769-P). | MSCs, SF, 3D, XF |
| 5 | Human Kidney Cancer Stem Cell Complete Growth Medium | Celprogen, Inc. (cat. no. M3611  7-44S) | Control medium for HKCSCs cells. Medium with fetal bovine serum, optimized for use with HKCSCs. Adherent; all cells grow well in this medium. | SCs, S, 2D, X* |
| 6 | Human Kidney Cancer Stem Cell Growth Medium | Celprogen, Inc. (cat. no. M36117-44) | Product the same as 5, but without serum. | SCs, SF, 2D, X* |
| 7 | Stemline® Mesenchymal Stem Cell Expansion Medium | Sigma Aldrich (cat. no. S1569-1L) | Medium promotes the optimal expansion of human MSCs from bone marrow. Contains serum. Adherent cell culture, the best results for Caki-1. | MSCs, S, 2D, X* |
| 10 | Mesenchymal Stem Cell Growth Medium | Promocell (cat. no. C-28010), currently not in stock; instead 28009 is provided as an updated version | Optimized formulation for routine culture of human MSCs. Adherent cell culture; fast confluence reach of all cell lines. | MSCs, RS, 2D, X |
| 11 | StemXVivo Serum-Free Tumorsphere Media + StemXVivo EMT Inducing Media Supplement | R&D Systems (cat. no. CCM012 + cat. no. CCM017) | Semi-solid medium formulated and optimized for tumorsphere formation, supplemented with epithelial to mesenchymal transition (EMT) inducing supplement. In Authors’ opinion one of the best medium since it results in large-surface aggregates recapitulating *in vivo* tumors. | SCs, SF, 3D, X* |
| 12 | MSCGM™ Mesenchymal Stem Cell Growth Medium | Lonza (cat. no. PT-3001) | A serum-containing medium designed to proliferate human bone marrow derived mesenchymal cells in an undifferentiated state; adherent cell culture. | MSCs, S, 2D, X* |
| 13 | Biowest RPMI 1640 | Biowest L0500 | RPMI are general purpose enriched media with extensive applications for a broad spectrum of mammalian and hybridoma cells including human myeloma, mouse hybridoma, human leukocytes, and B and T lymphocytes. It was originally formulated for suspension cultures and monolayer culture of human leukemic cells. | OCs, S, 2D, X* |
| 14 | NutriStem™ XF/FF Culture Medium for Human iPS and ES Cells | Stemgent (cat. no. 01-0005) | Xeno-free, feeder-free medium; contains no animal components. Low basic FGF (4 ng/ml), and TGFβ (<5 ng/ml). In Authors’ opinion, the only medium in which round-shaped sphere-like structures are formed (HKCSCs). | SCs (induced pluripotent stem cells) + OCs (human embryonic cells), S (human serum albumin), 3D (colonies), XF |

**Figure S1.** Classification of the tested media according to the features: ‘stemness’characteristics for other cell types, other than mesenchymal stem cells (MSCs), MSCs; serum – serum-free (SF), reduced-serum (RS), serum-containing (S); 2D and 3D structures; xeno-components – xeno-free (no animal-derived component, but may contain human-derived components) and xeno - not xeno free (with animal-derived components).


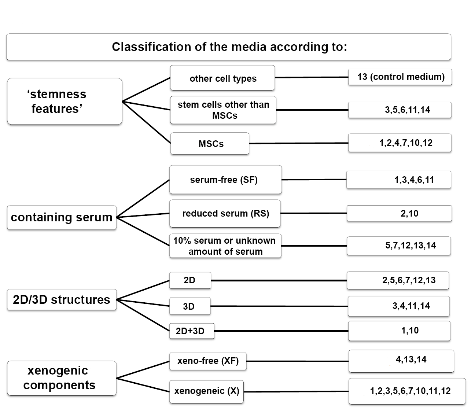


# RCC cell line growth in all analyzed media

**Table S4.** Cell lines and their media-induced growth characteristics.

| *Cell line* | *Origin* | *Company* | *Cell growth* | *Best for 3D structures* |
| --- | --- | --- | --- | --- |
| HKCSCs | Human Kidney Cancer Stem Cells | Celprogen Inc. (cat. no. 36117-44) | Fast confluence reach in media 1,2,5 and 7-14; 3D structures in media 1,9,11,14 | 14 |
| 769-P | Human renal cell carcinoma; primary tumor derived, *VHL* mutation | ATCC® (cat. no. CRL-1933™) | Fast confluence reach in media 2,5,7,10,12,13; 3D structures in media 1 and 4 | 4 |
| 786-O | Human renal cell carcinoma; primary tumor derived | ATCC® (cat. no. CRL-1932™) | Fast confluence reach in media 2,4,5,7,11,12,14; 3D structures present in media 11 and 14 | 14 |
| Caki-2 | Human renal cell carcinoma reported both as ccRCC and papillary RCC; primary tumor derived | ATCC® (cat. no. HTB-47™) | Fast confluence reach in media 1,2,4,5,7,8,10,12,13; 3D structures present in media 3 and 4 | 4 |
| ACHN | Human renal cell carcinoma; metastatic tumor derived (pleural effusion) | ATCC® (cat. no. CRL-1611™) | Fast confluence reach in media 2, 11-14; 3D structures present in media 11 and 14 | 4,11 |

**Fig S2.** Representative photos of cell morphology obtained in tested media and cells lines. When no growth was observed, photo was omitted.


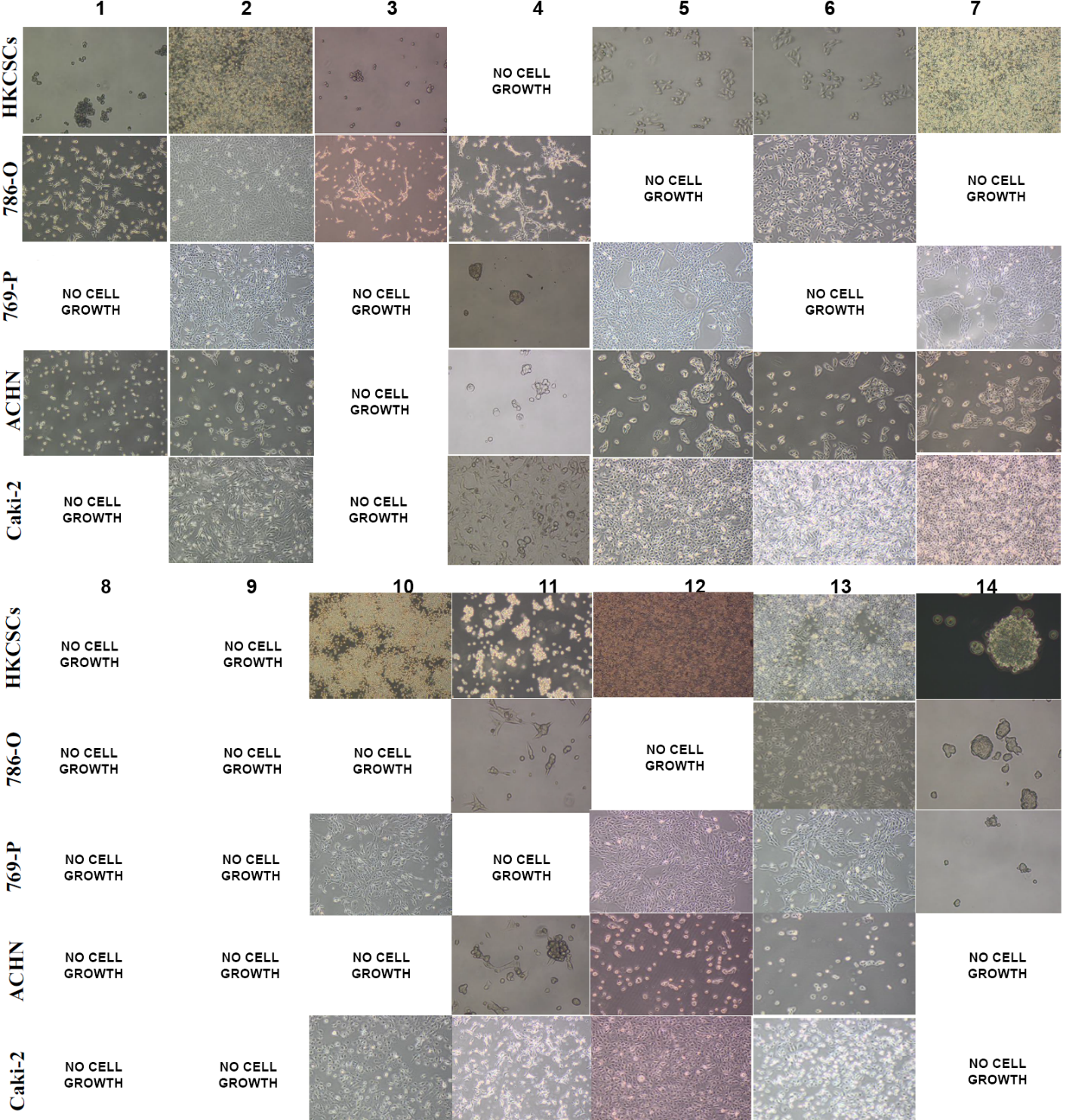


**Figure S3**. 2D and 3D growth of HKCSC cells cultured in most optimal experimental conditions: A) Medium 14 + Collagen, B) Medium 11 + Laminin, C) Medium 13 – control, D) Medium 14 + Laminin, E) Medium 11, F) Medium 5, G) Medium 14, H) Medium 9 + Laminin, I) Medium 9

**
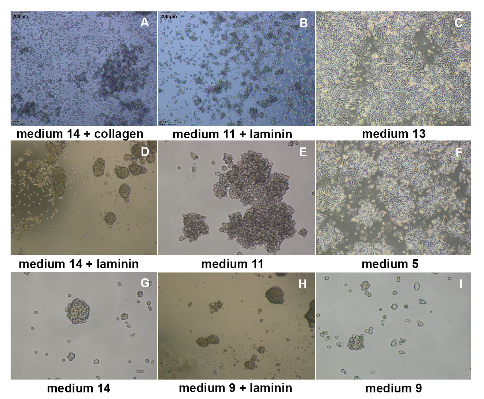
**

**Figure S4.** The size of three-dimensional structures measured with CellSens software (Olympus); area of representative aggregates/colonies/spheres was marked and calculated accordingly to magnification of the microscope. From the left to the right: from the structure of the largest to the smallest size (a). The graph illustrating average 3D structure sizes in specific cell line/media is located on the top of the figure, error bars as SD. Below the photographs of cells accordingly with the graph. The size was estimated in $\mu m$^3.^

^
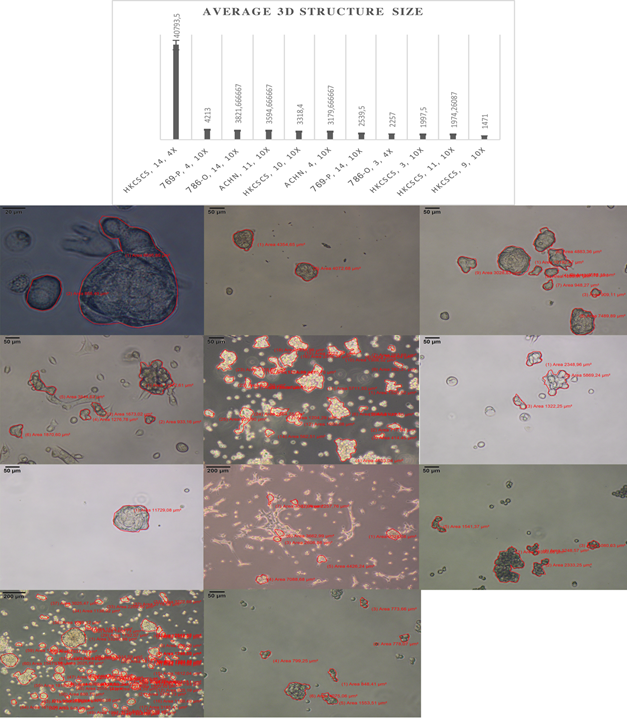
^

# RCC cell line drug induced growth inhibition testing

**Figure S5.** Drug response of RCC and HKCSC cells: A) 786-O in RPMI, B) 786-O in RPMI with sunitinib, C) 786-O in RPMI with sorafenib, D) 786-O in HKCSC medium, E) 786-O in HKCSC medium with sunitinib, F) 786-O in HKCSC medium with sorafenib, G) HKCSC in RPMI, H) HKCSC in RPMI with sunitinib I) HKCSC in RPMI with sorafenib, J) HKCSC in HKCSC medium, K) HKCSC in HKCSC medium with sunitinib L) HKCSC in HKCSC medium with sorafenib.


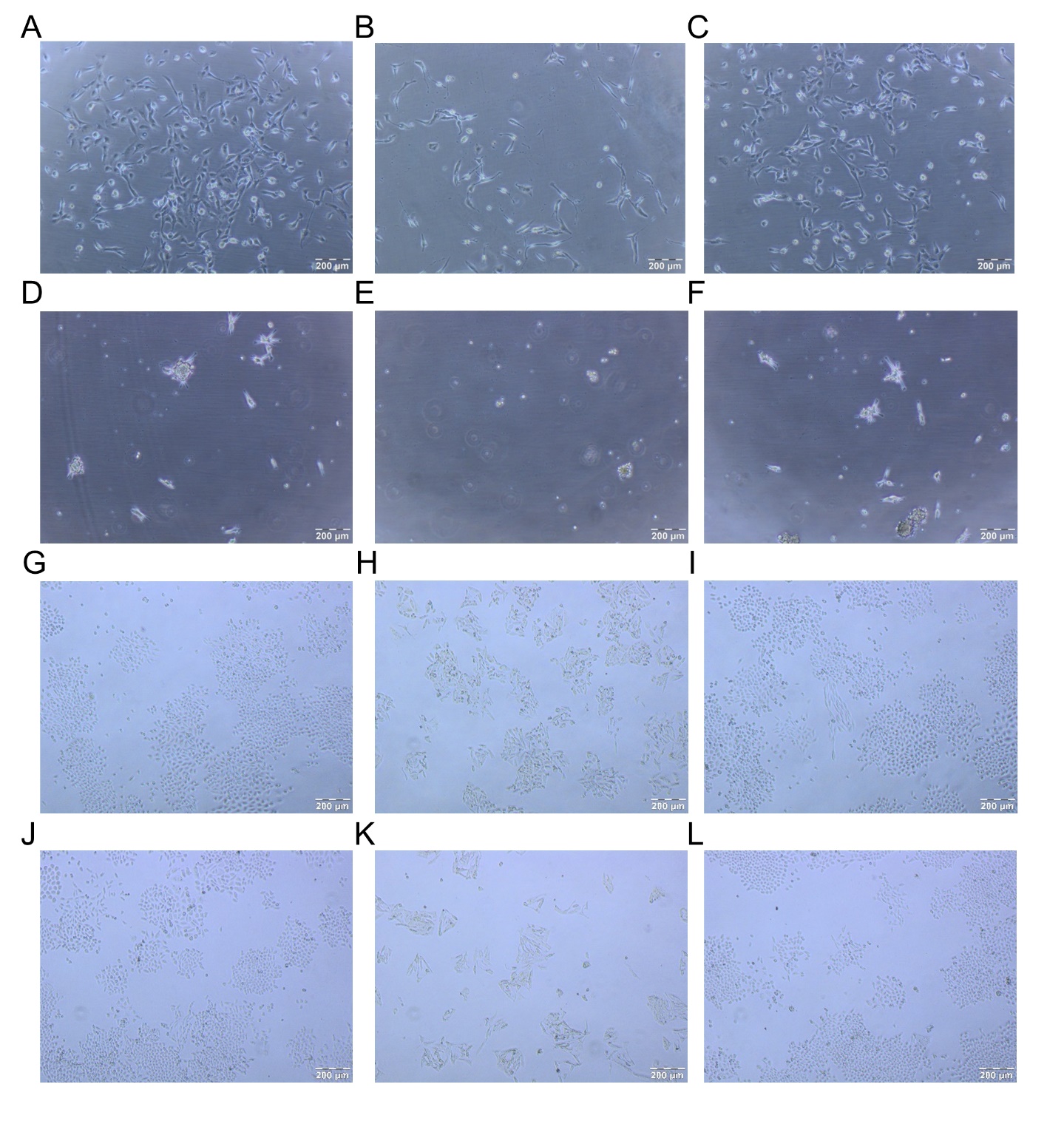


**Figure S6.** Time dependent 786-0 cells growth inhibition by sunitinib.


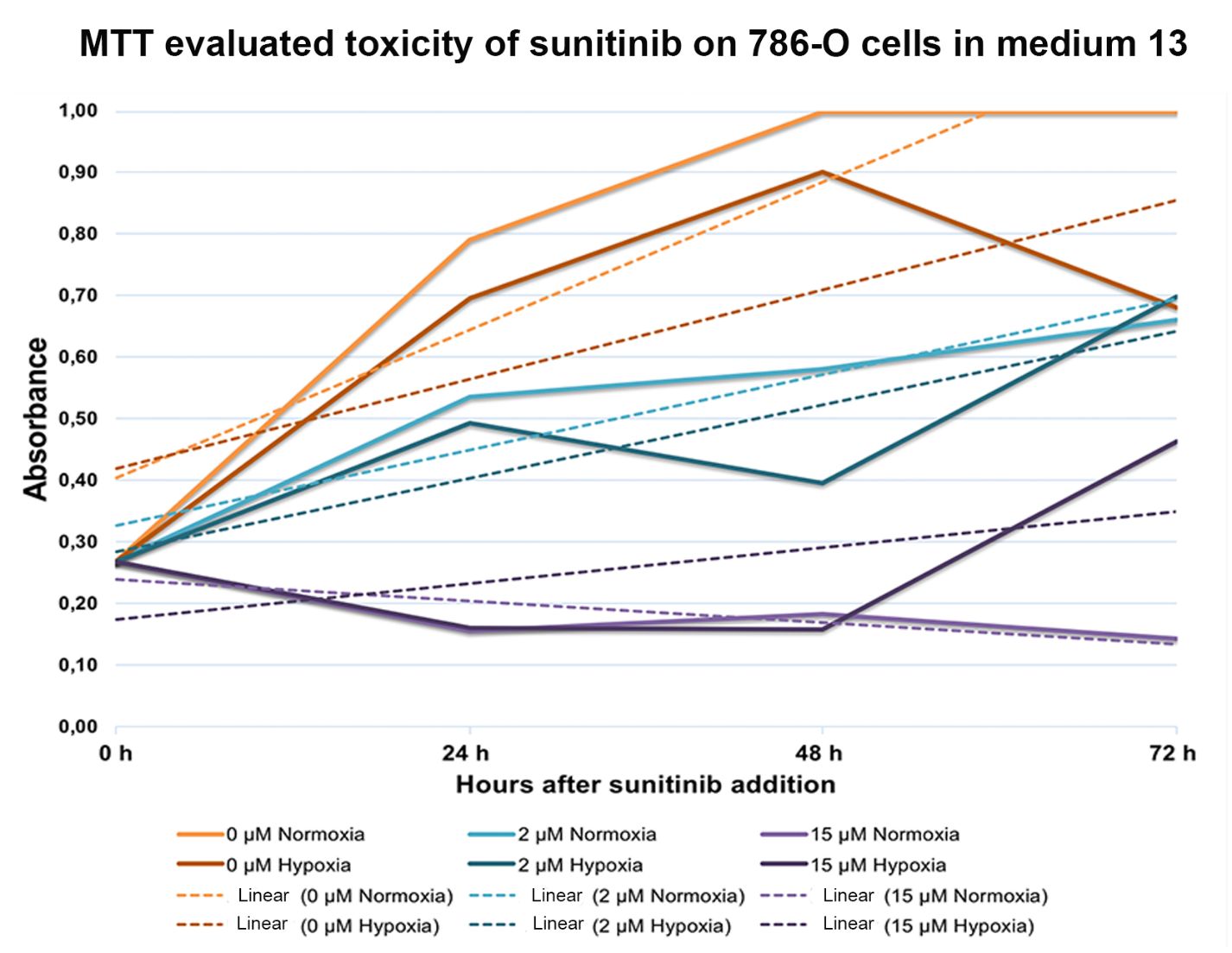


**Figure S7.** Oxygen tension dependent 786-0 cells growth inhibition by sorafenib in A) normoxia and B) hypoxia.


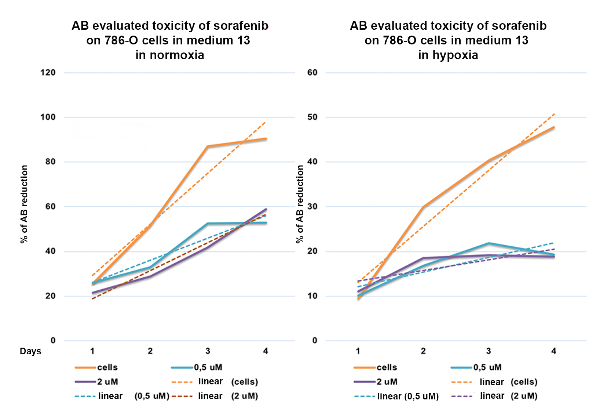

Supplement: Supplementary file 1 — Supplementary material 1 (DOCX 4854 kb) [file 10616_2018_273_MOESM1_ESM.docx]
